# Hereditary Elliptocytosis with Pyropoikilocytosis

**DOI:** 10.4274/tjh.2015.0054

**Published:** 2016-02-17

**Authors:** Turan Bayhan, Şule Ünal, Fatma Gümrük

**Affiliations:** 1 Hacettepe University Faculty of Medicine, Department of Pediatric Hematology, Ankara, Turkey

**Keywords:** Anemia, Elliptocytosis, Pyropoikilocytosis

A 17-day-old boy was admitted because of jaundice and anemia. He was born weighing 2900 g subsequent to a term gestation as the fourth child of first-degree cousin parents. The previous history revealed the administration of phototherapy for 4 days starting from the first day of life. Complete blood count revealed hemoglobin (Hb) of 6.9 g/dL, hematocrit of 19.8%, mean corpuscular volume (MCV) of 87.5 fL, red cell distribution width (RDW) of 37%, white blood cell count of 11.4x109/L, and platelet count of 263x109/L. Corrected reticulocyte count was 5.3%. Peripheral blood smear revealed polychromasia and pyropoikilocytosis. Direct antibody test was negative. Erythrocyte glucose-6-phosphate dehydrogenase, pyruvate kinase, and pyrimidine 5’ nucleotidase levels were normal. An erythrocyte transfusion was administered with a diagnosis of non-immune hemolytic anemia and the patient was discharged at the 26th day of life with initiation of folic acid. During his outpatient follow-up, he required erythrocyte transfusions 2 more times and the last transfusion was performed when he was 3 months old. At a visit 3 months after the last transfusion, his blood count was as follows: Hb of 9.5 g/dL, hematocrit of 28.2%, MCV of 68.2 fL, and RDW of 30.5%. Erythrocyte osmotic fragility was found to be normal and Hb electrophoresis revealed Hb F of 6.6% and Hb A2 of 1.7%. Upon physical examination he had mild jaundice and no splenomegaly. The parents’ blood counts were within normal ranges. Peripheral blood smear revealed prominent elliptocytes and occasional microcytic and fragmented erythrocytes with poikilocytosis (Figure 1). The clinical findings and laboratory results were diagnostic for the hereditary pyropoikilocytosis (HPP) type of hereditary elliptocytosis (HE), but in vitro fragmentation testing was not performed.

HE is a common hemolytic red cell membrane disease with variant clinical presentations [1]. Common mutations that cause HE are found in the α-spectrin, β-spectrin, and protein 4.1 genes [2]. The majority of patients with HE are asymptomatic, but HPP is a severe form of HE that presents with hemolytic anemia and jaundice during the infantile period. Erythrocyte morphology in HPP resembles that of blood smears in thermal burns with poikilocytes, red blood cell fragments, microspherocytes, and elliptocytes [3]. Low MCV (25 to 75 fL) due to fragmented red blood cells is characteristic and osmotic fragility is commonly normal [1,3].

## Figures and Tables

**Figure 1:**
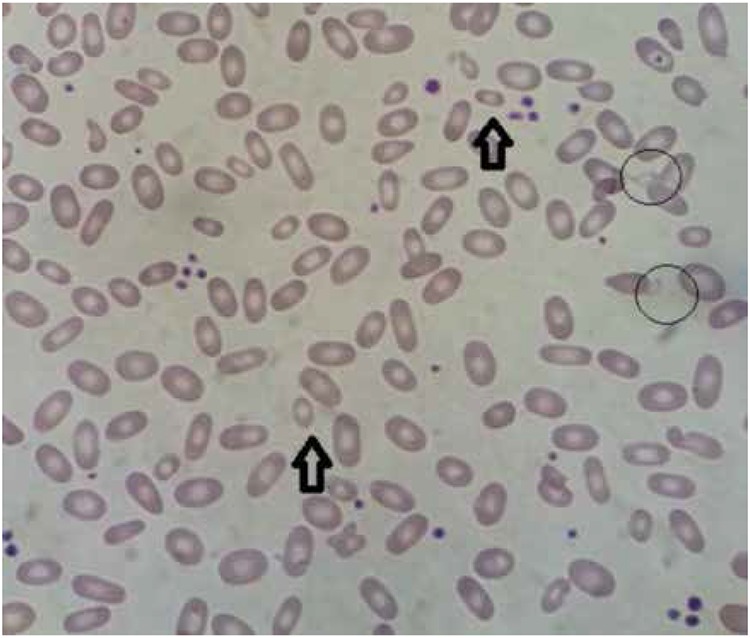
The peripheral blood smear of the patient: prominent elliptocytic erythrocytes, in addition to microcytic erythrocytes (arrows) and fragmented erythrocytes (circles).
